# Triterpene tales: Two genes involved in *Nicotiana attenuata* herbivore defense

**DOI:** 10.1093/plphys/kiae003

**Published:** 2024-01-08

**Authors:** Henryk Straube

**Affiliations:** Assistant Features Editor, Plant Physiology, American Society of Plant Biologists; Department of Plant and Environmental Sciences, Faculty of Science, Section for Plant Biochemistry, University of Copenhagen, 1871 Frederiksberg C, Copenhagen, Denmark

Plants are under constant threat from biotic stressors and have developed an impressive arsenal of specialized metabolites to defend themselves. Triterpenes are a diverse group of such specialized metabolites that help plants to protect themselves. Organisms produce thousands of distinct triterpenoids, with structures ranging from simple to complex (Thimmappa et al. 2014). The first committed step of triterpene biosynthesis in plants involves the cyclization of 2,3-oxidosqualene, which is catalyzed by enzymes called oxidosqualene synthases (OSCs; Noushahi et al. 2022). The majority of OSCs characterized from plants produce a single class of triterpenoid, although there are reports of OSCs being less product specific (Andre et al. 2016; Srisawat et al. 2019).

Triterpenes show a broad spectrum of biological activities against bacteria, fungi, or insects, and preventing animals from eating plants (Morrissey and Osbourn 1999; Tian et al. 2021). A well-known and widely used complex triterpene from plants is Azadirachtin, which shows insecticidal properties and is isolated from the neem tree (*Azadirachta indica*) (Dawkar et al. 2019). The compound is the active ingredient of many commercially available pesticides.

To study the interaction of specialized metabolites and the plant-herbivore interaction in general, scientists often use *Nicotiana attenuata*, which is commonly called coyote tobacco, as an ecological model plant. Coyote tobacco is native to western North America and is a host to various microorganisms and insects in its habitat. Using *N. attenuata*, the ecological roles of several specialized metabolites such as acyl sugars or nicotine in plant-herbivore interactions were uncovered (Weinhold and Baldwin 2011; Kumar et al. 2014). Interestingly, the triterpene metabolism and composition in *N. attenuata* was unclear.

In this issue of *Plant Physiology*, Yang et al. (2023) used phylogenetics, transient gene expression in *Nicotiana benthamiana*, and virus-induced gene silencing in combination with different metabolite analysis strategies to identify and characterize 2 OSCs from *N. attenuata*. They provide evidence for the involvement of these genes in plant-herbivore interactions and studied the triterpene composition in *N. attenuate* (Fig. 1).

**Figure 1.**
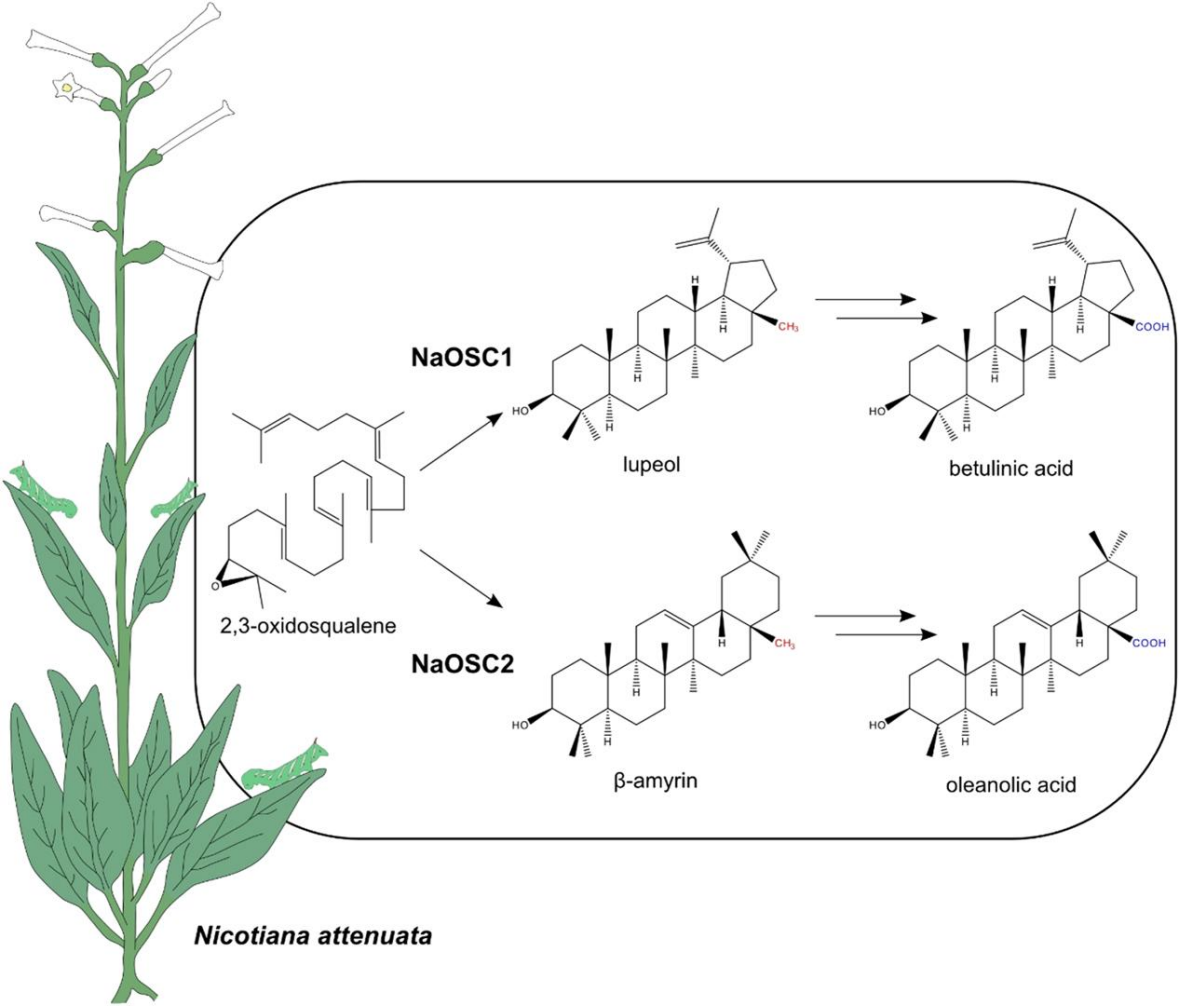
Synthesis of lupeol and *β*-amyrin from 2,3-oxidosqualene in *Nicotiana attenuata* by NaOSC1 and NaOSC2, resepctively. Lupeol is shown representatively as one of the 10 primary products of NaOSC1. All shown compounds, lupeol, *β*-amyrin, as well as their oxidized downstream products betulinic acid and oleanolic acid, reduce the mass of *Manduca sexta* larvae in feeding assays.

Using homology comparison, the authors identified 6 candidate genes encoding potential OSCs in the *N. attenuata* genome and named them *NaOSC1* to *NaOSC6*. Sequence analysis revealed that *NaOSC3* and *NaOSC6* are shorter and were thus excluded from further analyses. *NaOSC1* and *NaOSC2* shared high sequence similarity with known *β*-amyrin synthases, whereas *NaOSC4* and *NaOSC5* shared higher similarity with OSCs involved in lanosterol or cycloartenol biosynthesis.

The researchers expressed the 4 candidate genes in *N. benthamiana* and analyzed metabolites using gas chromatography mass spectrometry. Leaves overexpressing *NaOSC1* contained an increased amount of *β*-amyrin. Interestingly, the authors identified 10 new metabolites in plants overexpressing *NaOSC1*. Based on retention times and mass spectra of the new compounds, the authors propose that 7 of these new compounds are triterpenes. Leaves overexpressing *NaOSC2* showed no new peaks but contained an 80-fold higher amount of *β*-amyrin compared with leaves overexpressing *NaOSC1*. Overexpression of *NaOSC4* and *NaOSC5* led to neither new peaks nor an increased amount of *β*-amyrin. These results indicate that NaOSC1 has a lower specificity, whereas NaOSC2 is highly specific and synthesizes exclusively *β*-amyrin.

Using gas chromatography mass spectrometry, the scientists analyzed the content of primary products of NaOSC1 and NaOSC2 in different tissues of *N. attenuata*. The simple triterpenes were more abundant in younger tissues, including seedlings and young roots, consistent with the observed higher expression level of *NaOSC1* and *NaOSC2* in roots, flowers, and trichomes. The expression of these genes could also be increased by treating plants with the defense phytohormones methyl jasmonate. Interestingly, upon infestation with *Manduca sexta* larvae, the only *OSC* gene with a significant increase in expression was *NaOSC1*.

To confirm a potential involvement of *NaOSC1* and *NaOSC2* in the defense against *M. sexta* larvae, the authors performed virus-induced gene silencing to decrease the expression of the 2 genes. Silencing either of the 2 resulted in an increased susceptibility of *N. attenuata* to *M. sexta* larvae infestation. In vitro toxicity assays demonstrated that the immediate derivatives of NaOSC1 and NaOSC2—lupeol and *β*-amyrin—and their oxidized derivatives, betulinic acid and oleanolic acid, all resulted in diminished larval growth.

Although the transcripts of *NaOSC1* and *NaOSC2* were high in trichomes and responded to methyl jasmonate, the researchers were unable to detect the direct products in leaves of *N. attenuate*. A potential reason for this could be that the simple triterpenes do not accumulate as aglycones, or they are metabolized to more complex metabolites. This question was addressed by performing an untargeted metabolomic analysis of leaves from both control plants and leaves that underwent silencing of *NaOSC1* or *NaOSC2*. The authors putatively annotated 78% of features and noted that several of the those annotated as triterpenes showed a significant negative correlation with larval mass. These results indicate that the increased susceptibility of *N. attenuata* plants with silenced *NaOSC1* and *NaOSC2* could be a result of changes in the abundance of downstream complex triterpenoids.

In summary, Yang and colleagues (2023) identified and characterized NaOSC1 and NaOSC2, 2 enzymes catalyzing the first committed step of triterpenoid biosynthesis in *N. attenuata*. They showed the important role of these enzymes and their downstream products in the defense mechanism against *M. sexta* larvae.

## Dive Curated Terms

The following phenotypic, genotypic, and functional terms are of significance to the work described in this paper:

lupeol CHEBI: CHEBI:6570

triterpenes CHEBI: CHEBI:35191
